# The DNMT1/miR-34a/FOXM1 Axis Contributes to Stemness of Liver Cancer Cells

**DOI:** 10.1155/2020/8978930

**Published:** 2020-04-05

**Authors:** Xiaocheng Cao, Lihua Liu, Xiaozheng Cao, Yinghong Cui, Chang Zou, A. Chen, Yebei Qiu, Meifang Quan, Kaiqun Ren, Xiangding Chen, Jianguo Cao

**Affiliations:** ^1^Key Laboratory of Study and Discover of Small Targeted Molecules of Hunan Province, Medical College, Hunan Normal University, Changsha, Hunan 410013, China; ^2^Department of Pharmaceutical Science, Medical College, Hunan Normal University, Changsha, Hunan 410013, China; ^3^Laboratory of Molecular and Statistical Genetics, College of Life Sciences, Hunan Normal University, Changsha, Hunan 410081, China; ^4^Pharmacy Department, The Second Clinical Medical School of Jinan University, Shenzhen People's Hospital, Shenzhen 518020, China; ^5^Shenzhen Public Service Platform on Tumor Precision Medicine and Molecular Diagnosis, Shenzhen People's Hospital, Shenzhen 518020, China; ^6^Clinical Medical Research Center, The Second Clinical Medical School of Jinan University, Shenzhen People's Hospital, Shenzhen 518020, China

## Abstract

**Background:**

Whether DNA methyltransferase 1 (DNMT1)/miR-34a/FoxM1 signaling promotes the stemness of liver cancer stem cells (LCSCs) remains unclear. This study aimed to assess whether methylation-based silencing of miR-34a by DNMT1 contributes to stemness features via FoxM1 upregulation in LCSCs.

**Methods:**

The CD133^+^ subgroup of MHCC97H cells sorted by MACS was used as LCSCs. *DNMT1*, *BMI1*, *SOX2,* and *OCT4* mRNA levels, and *miR-34a* amounts were determined by qRT-PCR. DNMT1, CD44, and FoxM1 proteins were analyzed by immunoblot. Sphere and colony formation abilities were detected by respective assays. CD133^+^ cell percentages were assessed by flow cytometry. In vivo oncogenicity was evaluated using a tumor xenograft model in mice. The effects of DNMT1/miR-34a signaling on the stemness of LCSCs were examined by knockdown or overexpression of *DNMT1* and/or transfection of *miR-34a* mimic or inhibitor using lentivirus-delivery systems. FoxM1 association with miR-34a was detected by a reporter assay.

**Results:**

We here showed that LCSCs exhibited elevated DNMT1 activity and expression, lower miR-34a expression with higher promoter methylation, and stronger stemness, compared with the parental liver cancer cells. DNMT1 knockdown repressed DNMT1, increased miR-34a amounts by promoter demethylation, and reduced stemness in LCSCs, whereas DNMT1 overexpression had the opposite effects in liver cancer cells. Transfection with miR-34a mimic repressed the stemness of LCSCs, while miR-34a inhibitor significantly downregulated miR-34a and enhanced stemness, without affecting DNMT1 in liver cancer cells. MiR-34a mimic rescued the effects of DNMT1 overexpression on the stemness of LCSCs, without affecting DNMT1 expression. Finally, FOXM1 was identified as a direct target by miR-34a in LCSCs.

**Conclusions:**

We revealed that aberrant activation of DNMT1 causes miR-34a promoter methylation and suppression, leading to FoxM1 upregulation by disinhibition and promotion of LCSC stemness. These findings suggest that blockage of DNMT1/miR-34a-mediated FOXM1 upregulation might suppress liver cancer by targeting LCSCs.

## 1. Background

Liver cancer represents the fifth common malignant tumor and the third cause of cancer-associated deaths worldwide [1], and liver cancer stem cells (LCSCs) are responsible for tumor initiation and progression [2, 3]. Therefore, investigation of the mechanisms which drive the initiation of liver cancer could promote the development of new treatment tools that specifically target LCSCs.

Recent studies have demonstrated that the epigenetic mechanism possesses a key function in cancer initiation by regulating stem cell features [4, 5]. DNA methyltransferase 1 (DNMT1) is fundamental in maintaining the DNA methylation state. Furthermore, recent studies have revealed that DNMT1 maintains promoter hypermethylation of tumor-suppressor microRNAs [4–7]. It has been reported that DNMT1 is important in maintaining cancer stem cells (CSCs) in a self-renewal capability and promoting tumorigenesis, for example, mammary CSCs [8] and leukemia stem cells [9]. Importantly, Peng et al. reported that DNMT1 repression could upregulate miR-34a to induce cytotoxicity and apoptosis in breast cancer cells [10]. Chamani et al. showed that DNMT1 inhibition increases miR-34a expression, resulting in reduced viability of both Hep G2 and Huh7 cells [11]. However, whether abnormal DNMT1 expression resulting in miR-34a downregulation induces and maintains LCSC features remains largely unclear.

Studies have suggested miRNAs could either suppress or promote tumor initiation and progression [12–14]. MiR-34a, an miRNA well-studied for its association with tumorigenesis, is commonly downregulated in multiple cancers, including osteosarcoma [15], colorectal carcinoma [16], prostate cancer, [17] and liver cancer [18]. A number of reports have demonstrated that reexpression of miR-34a reduces malignancy potential in many cancer cell types [15–17]. It is noteworthy that miR-34a inhibits cancer stem cells in osteosarcoma and colorectal cancer [15, 16]. However, miR-34a's function in regulating LCSC stemness and the underlying mechanisms deserve further investigation.

Genome-wide association studies have proposed several putative molecules for liver cancer prognosis, for example, Forkhead box M1 (FoxM1) [19], a major modulator of tumorigenesis in various cancers, including liver cancer [20–22]. In addition, elevated FoxM1 is tightly related to unfavorable prognosis in liver cancer [20]. Furthermore, a study by Xu et al. revealed a putative axis comprising miR-34a and FoxM1, which likely controls the outcome of liver cancer patients [23]. Meanwhile, elevated FoxM1 expression associated with miR-34a downregulation plays a crucial role in liver cancer progression [23]. However, it is currently unknown whether low miR-34a expression followed by FoxM1 overexpression participates in enriching LCSCs and promoting stemness features in liver cancer cells. This study aimed to assess whether methylation-based silencing of miR-34a by DNMT1 contributes to stemness features via FoxM1 upregulation in liver cancer cells.

## 2. Methods

### 2.1. Cell and Sphere Cultures

MHCC97H, SK-Hep-1, and Hep G2 cells from the Cell Bank of Chinese Academy of Sciences (Shanghai, China) were grown in Dulbecco's Modified Eagle's Medium (DMEM; GIBCO, USA) containing 10% fetal bovine serum (FBS, Gibco) at 37°C in a humid atmosphere with 5% CO_2_.

Stem cell medium (SCM) [24] was employed to obtain tumor spheres. Spheres of CD133^+^ MHCC97H cells were further assessed as LCSCs [25]. Spheres of unsorted SK-Hep-1 and Hep G2 cells were used as liver cancer stem-like cells (LCSLCs).

### 2.2. MACS

CD133^+^ cells were isolated from MHCC97H cells as described previously using CD133 MicroBeads (Miltenyi Biotec, Germany). Briefly, the cells were separated using a MACS LS column (Miltenyi Biotec) carrying CD133/1 antibody conjugated with microbeads (MiltenyiBiotec).

### 2.3. DNMT1 Activity Detection

DNMT1 activity in all nuclear extract samples was measured nonradioactively by using DNA Methyltransferase Activity/Inhibition Assay Kit as instructed by the manufacturer (Epigentek, Group, USA). Relative DNMT1 activity was normalized to that of MHCC97H (or SK-Hep-1 and Hep G2) cells or LCSCs (or LCSLCs).

### 2.4. Quantitative Real-Time RT-PCR

TRIzol universal reagent (Tiangen Biotech, China) was used for total RNA extraction from MHCC97H cells (1 × 10^6^) or LCSCs (1 × 10^6^); miRcute miRNA isolation Kit (Tiangen Biotech) was employed to prepare total microRNA.

For mRNA quantitation, total RNA (2 *μ*g) underwent transcription with SureScript™ First-Strand cDNA Synthesis Kit (GeneCopoeia Inc., USA). The primer sequences used to amplify cDNA are listed in Supplementary [Supplementary-material supplementary-material-1]. Amplification was performed at 95°C for 10 min, followed by 40 cycles of 95°C (30 sec), 55°C (30 sec), and 70°C (30 sec).

For the determination of microRNA amounts, total miRNA (2 *μ*g) was transcribed and amplified with All-in-One™ miRNA qRT-PCR Detection Kit based on TaqMan MicroRNA Assay (Applied Biosystems), as instructed by the manufacturer (GeneCopoeia Inc.). U6 served as a reference control. Data analysis was carried out by the 2^−ΔΔCt^ method.

### 2.5. Methylation Specific PCR (MSP)

Cellular DNA from MHCC97H cells (1 × 10^6^) or LCSCs (1 × 10^6^) was isolated with DNA-EZ Reagents V All-DNA-Out (Sangon Biotech, China). Genomic DNA was incubated with Methylamp One-Step DNA Modification Kit (Epigentek) based on the manufacturer's directions. PCR was carried out with HotStar Taq Polymerase (Qiagen, Germany), and methylated and unmethylated PCR primers for the miR-34a promoter (in Supplementary [Supplementary-material supplementary-material-1]) were provided by Sangon Biotech. The MSP products were visualized by 2.0% agarose gel electrophoresis.

### 2.6. Spheroid Formation Assay

MHCC97H cells (1 × 10^3^) or LCSCs (1 × 10^3^) were cultured for six days to obtain spheres. The sphere formation rate was determined as number of spheres formed/number of cells seeded (1000 cells per well in a 24-well plate) ×100% [24]. The volume of spheroid was estimated as follows: *V*=(4/3)*πR*^3^ [25]. Three independent assays were carried out.

### 2.7. Clonogenic Assay

DMEM supplemented with 0.8% agarose (Invitrogen) was added to six-well plates (1 ml). Then, 500 MHCC97H cells or LCSCs in SCM containing 0.4% agarose (top layer) were plated per well in 24-well plates, for a three week-incubation. Colonies were counted under an inverted microscope (Olympus IX53, Japan). The colony formation rate was calculated as number of colonies formed/number of cells seeded (500 cells per well in a 24-well plate) ×100% [24]. Three independent assays were carried out.

### 2.8. Analysis of CD133 Expression

William's E medium supplemented with 20% FBS was used to incubate MHCC97H cells (1 × 10^6^) or LCSCs (1 × 10^6^) for 30 min; then, the cells were washed with PBS and incubated with PE-conjugated anti-CD133 or isotype control IgG2b (BioLegend) for 30 min at 4°C in the dark. A FACS Calibur™ system (BD, USA) was used to analyze the CD133^+^ cell population.

### 2.9. Western Blot Analysis

MHCC97H cells (1 × 10^6^) or LCSCs (1 × 10^6^) underwent lysis with chilled RIPA buffer (Beyotime Biotechnology, China). Proteins (40 *μ*g) determined by the Bradford assay (Bio-Rad, USA) were resolved by 10% SDS-PAGE and electrotransferred onto polyvinylidene fluoride (PVDF) membranes (Millipore, USA). After blocking (5% BSA in TBST for 2 h at room temperature) the membranes underwent overnight incubation in presence of primary antibodies targeting DNMT1 (Cell Signaling, USA), CD44 (Cell Signaling), FoxM1 (Santa Cruz, USA), and *β*-actin (Sigma-Aldrich) at 4°C. Appropriate HRP-conjugated secondary antibodies (Beyotime Biotechnology) were used to incubate the membranes for 1 h. Detection was performed with the enhanced chemiluminescence (ECL) kit (Amersham, USA).

### 2.10. Lentivirus Infection and miRNA Transfection

LV-15 (pGLVH1/RFP/Puro) lentiviral vectors carrying shRNA targeting human DNMT1 and LV8N (EF-1*α*F/mCherry/Puro) and lentiviral vectors overexpressing human DNMT1 were purchased from GenePharma (Shanghai). Target sequences of DNMT1 shRNAs were shDNMT1 #1: 5′GGAAATACTCCGACTACATCA3′; shDNMT1 #2: 5′GCGGCATGAACCGCTTCAATT3′; the scramble shRNA (sh NC) was 5′UUCUCCGAACGUGUCACGUAA3′. MHCC97H cells (1 × 10^5^) or LCSCs (1 × 10^5^) in the exponential growth phase underwent infection for 48 h with lentiviruses harboring DNMT1 shRNA, DNMT1 cDNA, and RFP constructs, respectively, in medium supplemented with 6 *μ*g/ml polybrene, followed by another 48 h of culture. Selection was performed under puromycin (5 *μ*g/ml) pressure for seven days, and further cell maintenance was carried out in presence of 1 *μ*g/ml puromycin.

micrON™ miR-34a mimic (50 nM) and micr*OFF*™ miR-34a inhibitor (100 nM) were transfected into MHCC97H cells or LCSCs with ibo*FECT*™ CP Reagent as directed by the manufacturer (RiboBio, Guangzhou, China). Meanwhile, the negative controls of miR-34a mimic (miR-NC) and miR-34a inhibitor (anti-NC) were transfected as described above for miR-34a mimic/inhibitor. Cells were submitted to incubation with small RNA complexes for 2 h before medium change.

Santa Cruz Biotechnology provided the control shRNA (sh NC) and FOXM1 shRNA (shFoxM1). The pcDNA3.1-Control (pcDNA) and pcDNA3.1-FOXM1 (pcDNA-FOXM1) were provided by Invitrogen. Lipofectamine™ 2000 (Invitrogen, CA, USA) was used for cell transfection in opti-MEM following the manufacturer's instructions.

### 2.11. In Vivo Tumorigenicity Experiments

Male nude BALB/c mice (4-5 weeks, 12–14 g) were provided by Hunan SJA Laboratory Animal Co., Ltd. (certificate No: 430047 00050992, Changsha, China). All experiments had approval from the Ethics Committee of Hunan Normal University and the Committee of Experimental Animal Feeding and Management (Permit No: 2018-050). Animal acclimation was performed for a week before experiments started.

MHCC97H cells stably expressing red fluorescent protein (1 × 10^5^) were subcutaneously injected into the left flank of mice (*n* = 6), with the corresponding LCSCs (1 × 10^3^) in the right flank, respectively, for in vivo tumorigenicity assays. Mice were sacrificed, and xenografts were harvested and weighed two months after injection.

For evaluating in vivo effect of DNMT1 on LCSC xenograft growth, the animals were administered subcutaneous injection of a 100 *μ*L mixture composed of LCSCs stably expressing red fluorescent protein (1 × 10^5^ cells) transduced with sh NC or shDNMT1 in DMEM/F12 medium without serum and matrigel (1 : 1; BD Biosciences), respectively, with three mice at six sites (*n* = 6) in each group.

To assess in vivo miR-34a's effect on LCSC xenograft growth, mice were subcutaneously injected with a 100 *μ*L mixture composed of LCSCs stably expressing red fluorescent protein (1 × 10^5^ cells) in DMEM/F12 medium without serum and matrigel (1 : 1; BD Biosciences). After xenograft volumes reached 200 mm^3^, the experimental group underwent intratumoral injection of 1 nmol micrON™ agomir-34a (RiboBio Co., Ltd., China) in 50 *μ*l phosphate-buffered saline (PBS), once weekly for three times in all; micrON™ agomir-NC was used in the control group. A total of three mice were injected at six sites (*n* = 6) per group. For in vivo imaging, the mice were anesthetized by isoflurane (RWD Life Science Co, Shenzhen, China) inhalation. Tumor volumes were assessed on an IVIS Lumina III in vivo Imaging System (Perkin Elmer, USA) and photographed (shooting mode, fluorescence; peak excitation wavelength, 587 nm; peak emission wavelength, 610 nm; exposure time, 0.1 s). Fluorescence intensity was recorded and analyzed with the living image in vivo imaging software (PerkinElmer) at the end of the experiment. After euthanasia (CO_2_ inhalation), the xenografts were obtained, weighed, and snap-frozen (liquid nitrogen) or fixed with 10% formalin for subsequent assays.

### 2.12. Immunohistochemical Staining

Immunohistochemical staining was performed according to standard procedures. Tissue slides underwent incubation at 4°C overnight with anti-CD133, anti-CD44, and anti-DNMT1 antibodies (1 : 200; Cell Signaling Technology). PBS was used instead of primary antibodies as a negative control. IHC staining was quantitated by the IHC toolbox of Image J software (version 1.50i) [26].

### 2.13. Luciferase Assay

The wild-type FOXM1 3′UTR target sequence comprising the miR-34a binding site was inserted in the pLUC luciferase vector (Ambion, USA), as directed by the manufacturer. A mutated FOXM1 3′UTR sequence served as a control. The above constructs (0.2 *μ*g) were, respectively, transfected into MHCC97H cells or LCSCs in 24-well microplates alongside miR-34a or miR-NC with Lipofectamine 3000 for 48 h. Then, the dual luciferase reporter assay system was used to assess luciferase activity, as directed by the manufacturer (Promega).

### 2.14. Statistical Analysis

The SPSS 20.0 software (IBM, Armonk, NY, USA) was employed for statistical analysis. Data are mean ± standard deviation (SD). Comparisons to the control groups were performed by two-tailed Student's *t*-test. One way ANOVA with the Tukey's post-hoc test was employed for pairwise comparisons among multiple groups. *P* < 0.05 was considered statistically significant.

## 3. Results

### 3.1. DNMT1 Activation Promotes the Stemness of LCSCs

**Figure 1:**
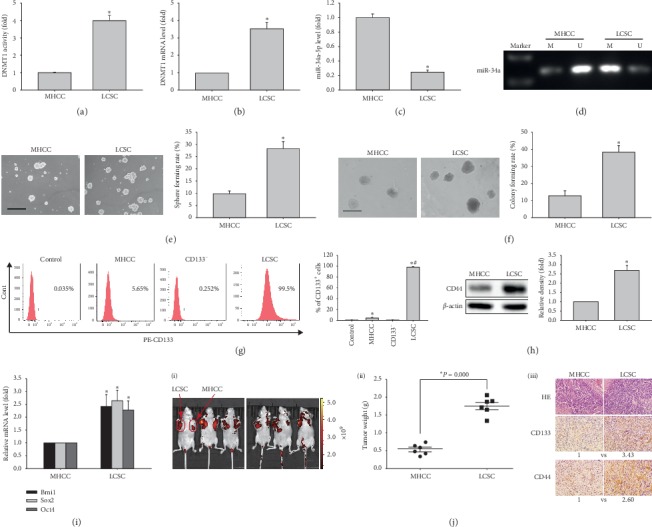
Stemness comparison between MHCC97H cells and LCSCs. (a) and (b) DNMT1 activities and mRNA amounts, respectively, in MHCC97H cells and LCSCs. (c) miR-34a-5p levels in MHCC97H cells and LCSCs. (d) miR-34a promoter methylation levels in MHCC97H cells and LCSCs. ((e) and (f)) Representative images of spheres and colonies (left) (scale bar, 200 *μ*m); sphere formation efficiencies and colony formation rates (right) in MHCC97H cells and LCSCs. (g) CD133 expression levels in MHCC97H cells and LCSCs. (h) CD44 protein amounts in MHCC97H cells and LCSCs. (i) *Bmi1*, *Sox2,* and *Oct4* mRNA amounts in MHCC97H cells and LCSCs. ^*∗*^*P* < 0.05 vs. MHCC97H cells (*n* = 3). (j) In vivo carcinogenicity in MHCC97H cell and LCSC xenograft models in nude mice, including lesion size (i), weight (ii), and histology and expression of the CD133 and CD44 proteins (iii). ^*∗*^*P* < 0.05 vs. MHCC97H cells (*n* = 6).

DNMT1 is important in maintaining cancer stem cells since upregulated miR-34a associated with DNMT1 inhibition can reduce the viability of liver cancer cells [27]. We therefore initially explored whether CD133^+^ spheres from MHCC97H cells used as LCSCs are involved in DNMT activation and methylation silencing of miR-34a. Compared with MHCC97H cells, DNMT1 activity and mRNA amounts were elevated in LCSCs (Figures 1(a) and 1(b)). We found that DNMT1 activation downregulated miR-34a and enhanced mir-34a promoter methylation in LCSCs (Figures 1(c) and 1(d)). In functional assays, sphere formation ability and clonogenicity showed significant increases in LCSCs compared with MHCC97H cells (Figures 1(e) and 1(f)). In addition, sphere size was larger in LCSCs than MHCC97H cells (Supplementary [Supplementary-material supplementary-material-1]). Furthermore, CD133^+^ cells and CD44 protein expression were increased in LCSCs compared with MHCC97H cells (Figures 1(g) and 1(h)). In addition, *BMI1*, *SOX2,* and *OCT4* (Figure 1(i)) mRNA amounts in LCSCs were higher than those of MHCC97H cells. To determine the ability of sphere cultures to maintain the nature of LCSCs, CD133^+^ cells sorted from the MHCC97H cell line underwent serial passage, and the CD133^+^ subpopulation was examined by flow cytometry. We found that high purity of CD133^+^ cells could be maintained from the first generation to the fourth (Supplementary [Supplementary-material supplementary-material-1]). Accordingly, CD133^+^ spheres in the fourth generation were assessed in this study. Finally, we also demonstrated that MHCC97H-derived CD133^+^ spheres had the LCSC characteristic of strong tumorigenicity in vivo (Figure 1(j) and [Supplementary-material supplementary-material-1]). These results corroborated our previous study [25].

**Figure 2:**
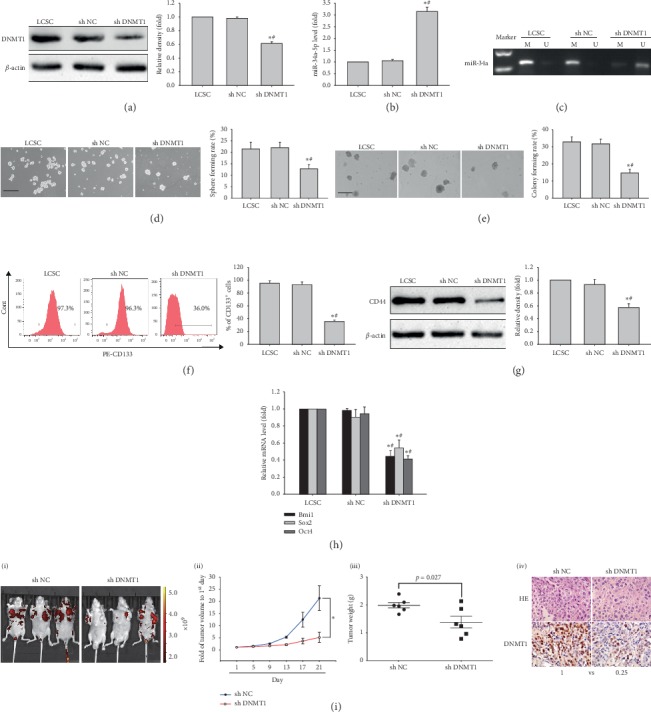
Effects of DNMT1 shRNA on stem-like features of MHCC97H-derived LCSCs. (a) DNMT1 protein amounts in LCSCs transfected with DNMT1 shRNA, with *β*-actin as a loading control. (b) miR-34a-5p levels in LCSCs transfected with DNMT1 shRNA. (c) miR-34a promoter methylation in LCSCs transfected with DNMT1 shRNA. ((d) and (e)) Representative images of spheres and colonies in LCSCs transfected with DNMT1 shRNA (left) (Scale bar, 200 *μ*m); sphere formation efficiencies and colony formation rates were determined (right). (f) CD133 expression in LCSCs transfected with DNMT1 shRNA. (g) CD44 protein amounts in LCSCs transfected with DNMT1 shRNA. (h) *Bmi1*, *Sox2,* and *Oct4* mRNA amounts in LCSCs transfected with DNMT1 shRNA. ^*∗*^*P* < 0.05 vs. LCSC (*n* = 3); ^#^*P* < 0.05 vs. LCSCs transfected with NC shRNA (*n* = 3). (i) Images of subcutaneous xenografts of LCSCs (1 × 10^5^) expressing red fluorescent protein (RFP) transfected with sh NC or DNMT1 shRNA; (ii) and (iii) comparison of tumor growth curves and weights of tumors from LCSCs expressing RFP transfected with sh NC and DNMT1 shRNA. ^*∗*^*P* < 0.05 vs. sh NC group (*n* = 6). (iv) Micrographs of H&E staining (×200) and immunohistochemistry (×400) obtained under an optical microscope.

To assess the role of DNMT1 activation in maintaining stemness in LCSCs, DNMT1 shRNA was used to knockdown *DNMT1* in LCSCs. The knockdown efficiency for *DNMT1* was higher in LCSCs transduced with shDNMT1 #2 compared with shDNMT1 #1 (Supplementary [Supplementary-material supplementary-material-1]). LCSCs transduced with shDNMT1 #2 were assessed in subsequent assays and termed shDNMT1. DNMT1 amounts at the protein level in LCSCs were remarkably reduced (Figure 2(a)). Meanwhile, miR-34a were increased with attenuated miR-34a-5p promoter methylation, compared with the sh NC control group and nontransduced cells (Figures 2(b) and 2(c)). DNMT1 knockdown in LCSCs significantly reduced sphere and colony formation rates (Figures 2(d) and 2(e)). Furthermore, the CD133^+^ cell population was decreased, as well as CD44 protein amounts, in LCSCs harboring DNMT1 shRNA (Figures 2(f) and 2(g)). In addition, *BMI1*, *SOX2,* and *OCT4* (Figure 2(h)) mRNA amounts in LCSCs harboring DNMT1 shRNA were lower than those of the sh NC control group and nontransduced cells. Most importantly, we demonstrated that DNMT1 knockdown significantly repressed tumor growth in nude mouse xenograft models (Figure 2(i)). Our results thus suggested that DNMT1 knockdown repressed stemness in LCSCs, possibly by upregulating miR-34a via promoter hypomethylation.

**Figure 3:**
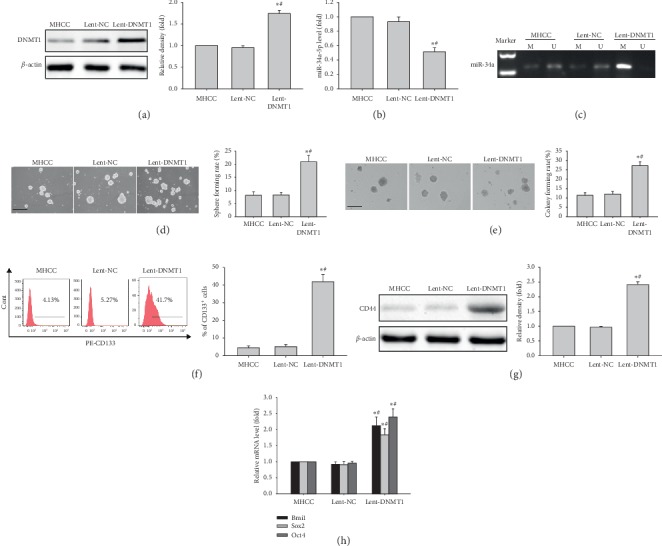
Effects of DNMT1 overexpression on stem-like features of MHCC97H cells. (a) DNMT1 protein amounts in MHCC97H cells transfected with Lent-DNMT1, with *β*-actin as a loading control. (b) miR-34a-5p levels in MHCC97H cells transfected with Lent-DNMT1. (c) miR-34a promoter methylation in MHCC97H cells transfected with Lent-DNMT1. (d, e) Representative images of spheres and colonies in MHCC97H cells transfected with Lent-DNMT1 (left) (scale bar, 200 *μ*m); sphere formation efficiencies and colony formation rates (right). (f) CD133 expression in MHCC97H cells transfected with DNMT1 cDNA. (g) CD44 protein expression in MHCC97H cells transfected with Lent-DNMT1. (h) *Bmi1*, *Sox2,* and *Oct4* mRNA amounts in MHCC97H cells transfected with Lent-DNMT1. ^*∗*^*P* < 0.05 vs. MHCC97H cells (*n* = 3);^#^*P* < 0.05 vs. MHCC97H cells transfected with Lent-NC.

MHCC97H cells were next infected with DNMT1-harboring lentiviruses (Lent-DNMT1), and the effect of DNMT1 overexpression on stemness was evaluated. As shown in Figure 3(a), DNMT1 amounts at the protein level showed a significant increase in MHCC97H cells expressing DNMT1 compared with the Lent-NC control group and nontransduced cells (MHCC). Meanwhile, miR-34a amounts were decreased, along with increased promoter methylation (Figures 3(b) and 3(c)). DNMT1 overexpression enhanced sphere and colony formation capabilities in MHCC97H cells compared with the Lent-NC control group and nontransduced cells (Figures 3(d) and 3(e)). Furthermore, the CD133^+^ cell population was increased, as well as CD44 protein amounts, in DNMT1 overexpressing MHCC97H cells (Figures 3(f) and 3(g)). In addition, *BMI1*, *SOX2,* and *OCT4* (Figure 3(h)) mRNA amounts in DNMT1 overexpressing MHCC97H cells were higher than those of the Lent-NC control group and nontransduced cells. Collectively, the above findings suggested DNMT1 activation could promote and maintain the stemness feature in LCSCs by downregulating miR-34a via promoter hypermethylation.

### 3.2. MiR-34a Overexpression Represses Stemness in LCSCs

**Figure 4:**
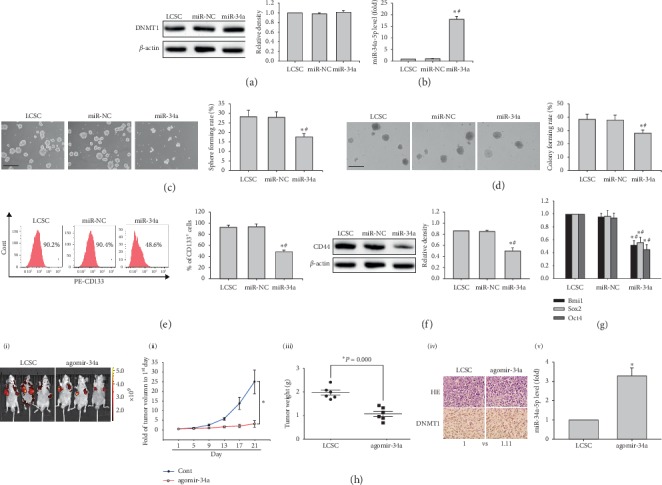
Effects of miR-34a mimic on stem-like features of MHCC97H derived LCSCs. (a) DNMT1 protein amounts in LCSCs transfected with miR-34a mimic. (b) miR-34a-5p levels in LCSCs transfected with miR-34a mimic. (c, d) Representative images of spheres and colonies in LCSCs transfected with miR-34a mimic (left) (scale bar, 200 *μ*m); sphere formation efficiencies and colony formation rates were determined (right). (e) CD133 expression in LCSCs transfected with miR-34a mimic. (f) CD44 protein amounts in LCSCs transfected with miR-34a mimic. (g) *Bmi1*, *Sox2,* and *Oct4* mRNA amounts in LCSCs transfected with miR-34a mimic. (h) Images of subcutaneous xenografts from LCSCs (1 × 10^5^) expressing red fluorescent protein (RFP) treated with agomir-NC or agomir-34a (i); comparison of tumor growth curves and weights of tumors from LCSCs expressing RFP treated with agomir*-*NC or agomir-34a (ii, iii). ^*∗*^*P* < 0.05 vs. transfected with agomir*-*NC (*n* = 6). (iv) Micrographs of H&E staining (×200) and immunohistochemistry (×400) obtained under an optical microscope. (v) miR-34a-5p levels in xenografts from LCSCs transfected with agomir-34a or agomir-NC. ^*∗*^*P* < 0.05 vs. LCSCs (*n* = 6); ^#^*P* < 0.05 vs. LCSCs transfected with agomir*-*NC (*n* = 6).

To assess in vitro miR-34a's effects on stemness in LCSCs, the latter cells underwent transfection with miR-34a mimic (miR-34a) or miR-NC. Although showing no effects on DNMT1 protein levels (Figure 4(a)), miR-34a was increased in LCSCs after transfection with miR-34a in comparison with the miR-NC transfection group or nontransfected cells (Figure 4(b)). Transfection with miR-34a reduced sphere and colony formation capabilities in LCSCs in comparison with the miR-NC transfection group or nontransfected cells (Figures 4(c) and 4(d)). Furthermore, the CD133^+^ cell population was decreased, and CD44 protein amounts were reduced by transfection of LCSCs with miR-34a (Figures 4(e) and 4(f)). *BMI1*, *SOX2,* and *OCT4* mRNAs (Figure 4(g)) were also reduced in LCSCs transfected with miR-34a. Most importantly, agomir-34a effectively inhibited tumor growth in nude mice bearing LCSCs (Figure 4(h)). These findings indicated that miR-34a reduced stemness in LCSCs, without affecting DNMT1 activity or expression, suggesting that DNMT1 might act upstream of miR-34a.

**Figure 5:**
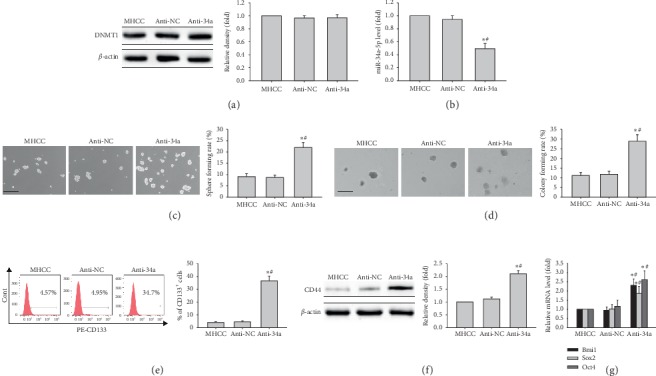
Effects of miR-34a inhibitor on stem-like features of MHCC97H cells. (a) DNMT1 protein amounts in MHCC97H cells transfected with miR-34a inhibitor. (b) miR-34a-5p levels in MHCC97H cells transfected with miR-34a inhibitor. (c, d) Representative images of spheres and colonies formed by MHCC97H cells transfected with miR-34a inhibitor (left) (scale bar, 200 *μ*m); sphere formation efficiencies and colony formation rates (right). (e) CD133 expression in MHCC97H cells transfected with miR-34a inhibitor. (f) CD44 protein amounts in MHCC97H cells transfected with miR-34a inhibitor. (g) *Bmi1*, *Sox2,* and *Oct4* mRNA amounts in MHCC97H cells transfected with miR-34a inhibitor. (*n* = 3); ^*∗*^*P* < 0.05 vs. MHCC97H cells (*n* = 3); ^#^*P* < 0.05 vs. MHCC97H cells transfected with miR-NC (*n* = 3).

MHCC97H cells were transfected with anti-34a and anti-NC, respectively, in loss of miR-34a function assays. MiR-34a expression was decreased in MHCC97H cells transfected with anti-34a compared with the anti-NC group or nontransfected cells, without affecting DNMT1 protein expression (Figures 5(a) and 5(b)). Transfection of MHCC97H cells with anti-34a increased sphere and colony formation capabilities (Figures 5(c) and 5(d)). Furthermore, the CD133^+^ cell population was increased, and CD44 protein amounts were elevated upon transfection of MHCC97H cells with anti-miR-34a (Figures 5(e) and 5(f)). In addition, *BMI1*, *SOX2,* and *OCT4* mRNAs (Figure 5(g)) were increased in MHCC97H cells transfected with anti-miR-34a. Collectively, the above findings suggested that miR-34a knockdown could facilitate stemness in LCSCs, without affecting DNMT1 expression, further demonstrating that DNMT1 was upstream of miR-34a.

**Figure 6:**
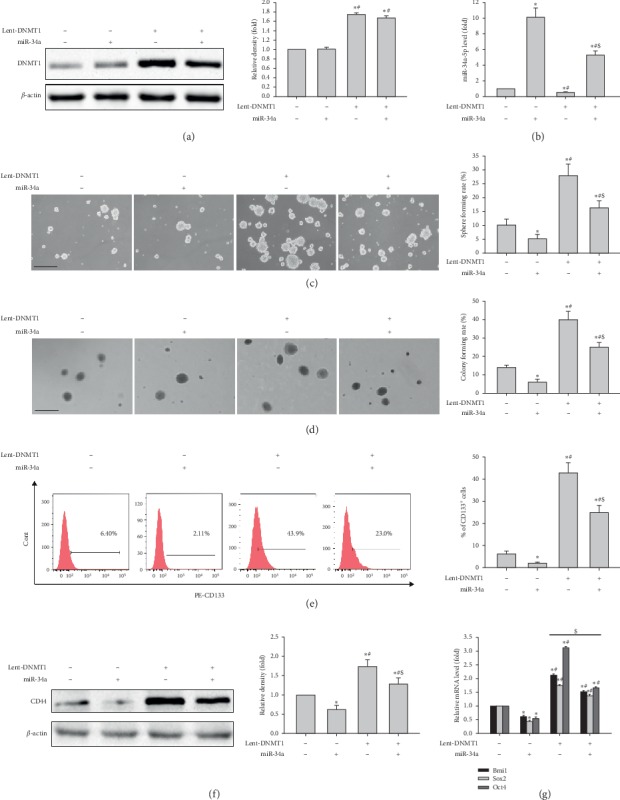
Effects of DNMT1 overexpression combined with miR-34a mimic on stemness of MHCC97H cells. (a) DNMT1 protein amounts in MHCC97H cells overexpressing DNMT1 after transfection with miR-NC or miR-34a mimic. (b) miR-34a-5p levels in MHCC97H cells overexpressing DNMT1 upon transfection with miR-NC or miR-34a mimic. (c, d) Representative images of spheres and colonies formed by MHCC97H cells overexpressing DNMT1 upon transfection with miR-NC or miR-34a mimic (left) (scale bar, 200 *μ*m); sphere formation efficiencies and colony formation rates (right). (e) CD133 expression in MHCC97H cells overexpressing DNMT1 upon transfection with miR-NC or miR-34a mimic. (f) CD44 protein amounts in MHCC97H cells overexpressing DNMT1 after transfection with miR-NC or miR-34a mimic. (g) *Bmi1*, *Sox2,* and *Oct4* mRNA amounts in MHCC97H cells overexpressing DNMT1 upon transfection with miR-NC or miR-34a mimic. ^*∗*^*P* < 0.05 vs. MHCC97H cells transfected with miR-NC (*n* = 3); ^#^*P* < 0.05 vs. MHCC97H cells transfected with miR-34a mimic (*n* = 3); *P* < 0.05 vs. MHCC97H cells transduced with DNMT1 cDNA.

To assess miR-34a's function in the inductive effects of DNMT1 overexpression on stemness in liver cancer cells, MHCC97H cells expressing DNMT1 were transfected miR-34a mimic.  Figure 6(a) shows that miR-34a had no effects on DNMT1 protein expression, either at baseline or after DNMT1 overexpression, in MHCC97H cells. Conversely, miR-34a transfection could abrogate miR-34a downregulation by DNMT1 overexpression (Figure 6(b)). DNMT1 overexpression enhanced sphere and colony formation capabilities in MHCC97H cells, and these effects were abrogated by transfection with miR-34a (Figures 6(c) and 6(d)). Furthermore, DNMT1 overexpression increased the CD133^+^ cell population and CD44 protein amounts; these effects were also abrogated by transfection with miR-34a (Figures 6(e) and 6(f)). In addition, DNMT1 overexpression increased *BMI1*, *SOX2,* and *OCT4* mRNAs, which were abrogated by transfection with miR-34a (Figure 6(g)). Together, the above findings suggested that miR-34a reversed the inductive effects of DNMT1 overexpression on stemness in liver cancer cells, without affecting DNMT1 expression, further confirming that DNMT1 was upstream of miR-34a.

### 3.3. FoxM1 Is a Direct miR-34a Target in LCSCs

**Figure 7:**
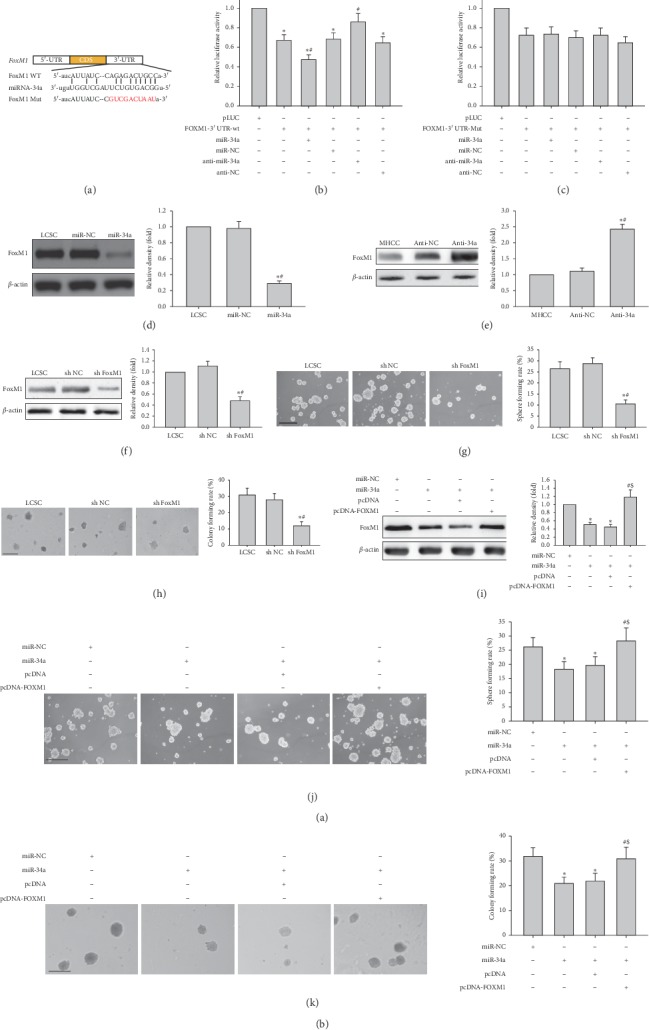
Sequence-specific downregulation of FOXM1 by miR-34a. (a) Wild type (WT) and mutated (Mut) 3′-UTRs of FOXM1 binding sites with miR-34a. (b, c) Luciferase activities in MHCC97H cells transfected with both firefly luciferase constructs comprising FOXM1-WT or FOXM1-Mut 3′-UTR and miR-34a mimic or the corresponding negative control (NC). (d) FOXM1 protein amounts in LCSCs upon transfection with miR-NC or miR-34a mimic. ^*∗*^*P* < 0.05 vs. LCSC (*n* = 3); ^#^*P* < 0.05 vs. LCSCs transfected with miR-NC (*n* = 3). (e) FOXM1 protein amounts in MHCC97H cells submitted to transfection with anti-NC or miR-34a inhibitor. ^*∗*^*P* < 0.05 vs. MHCC97H cells (*n* = 3); ^#^*P* < 0.05 vs. MHCC97H cells transfected with anti-NC (*n* = 3). (f) FOXM1 protein amounts in LCSCs submitted to transfection with FOXM1 shRNA. (g, h) Representative images of spheres and colonies formed by LCSCs transfected with FOXM1 shRNA (left) (scale bar, 200 *μ*m); sphere formation efficiencies and colony formation rates (right). ^*∗*^*P* < 0.05 vs. LCSC (*n* = 3); ^#^*P* < 0.05 vs. LCSCs transfected with sh NC (*n* = 3). (i) FOXM1 overexpression rescued FOXM1 downregulation at the mRNA and protein levels in LCSCs transfected with miR-34a mimic. (j, k) Representative images of spheres and colonies formed by LCSCs expressing miR-34a transfected with FOXM1 cDNA (left) (scale bar, 200 *μ*m); sphere formation efficiencies and colony formation rates (right). ^*∗*^*P* < 0.05 vs. LCSCs transfected with miR-NC (*n* = 3); ^#^*P* < 0.05 vs. LCSCs transduced with pcDNA (*n* = 3).

It was demonstrated that elevated FoxM1 expression associated with miR-34a downregulation has an important function in liver cancer progression [28]. In addition, we and others have shown that FOXM1 is a key regulator of stemness in various cancers, including liver cancer [29, 30]. We found that the 3′-UTR of human FOXM1 mRNA contains a possible miR-34a binding site by TargetScan (Figure 7(a)). To confirm this finding, pLUC/FOXM1-3′-UTR-wt and pLUC/FOXM1-3′-UTR-mut, respectively, were cloned downstream of the firefly luciferase reporter gene and then cotransfected with miR-34a mimic (miR-34a) or miR-34a inhibitor (anti-miR-34a) into MHCC97H cells, followed by luciferase activity determination. Luciferase activity was decreased by approximately 52.8% upon cotransfection of miR-34a mimic and pLUC/FOXM1-3′-UTR-wt (Figure 7(b)) and elevated by 34.2% (*P* < 0.05) after miR-34a knockdown (Figure 7(b)). Meanwhile, cotransfection of MHCC97H cells with pLUC/FOXM1-3′-UTR-mut and miR-34a mimic or miR-34a inhibitor did not affect luciferase activity (Figure 7(c)). We next assessed FoxM1 protein amounts in LCSCs and MHCC97H cells after transfection with miR-34a mimic (or miR-NC) or miR-34a inhibitor (or anti-NC) by immunoblot, respectively. MiR-34a reduced FoxM1 protein amounts in LCSCs (Figure 7(d)). Conversely, miR-34a knockdown resulted in significantly elevated FoxM1 protein amounts in MHCC97H cells (Figure 7(e)). The above findings indicated miR-34a negatively regulated FOXM1 via direct binding to the 3′-UTR of its mRNA in LCSCs and MHCC97H cells.

To investigate whether FOXM1 represents a functional miR-34a target for stemness acquisition in hepatic carcinoma, whether FOXM1 silencing could replicate the phenotypic effects of miR-34a overexpression was assessed. As shown in Figure 7(f), FOXM1 was successfully knocked down in shFOXM1-LCSCs, based on immunoblot data. As expected, sphere and colony formation capabilities were significantly reduced in shFOXM1-LCSCs compared with the sh NC control group or nontransfected cells (Figures 7(g) and 7(h)). Thus, FOXM1 knockdown could repress stemness in LCSCs, mimicking the effects of miR-34a upregulation.

We next overexpressed FOXM1 by introducing a plasmid carrying FOXM1 cDNA in LCSCs expressing miR-34a. Interestingly, FOXM1 overexpression rescued the reduction of FOXM1 protein by miR-34a in LCSCs (Figure 7(i)). FOXM1 cDNA transfection also rescued the sphere and colony formation abilities inhibited by miR-34a in LCSCs (Figures 7(j) and 7(k)). These findings indicated that FOXM1 reexpression rescued miR-34a-associated suppression of stemness in LCSCs.

### 3.4. The DNMT1/miR-34a/FoxM1 Axis Is a Novel Target for Stemness in LCSLCs

**Figure 8:**
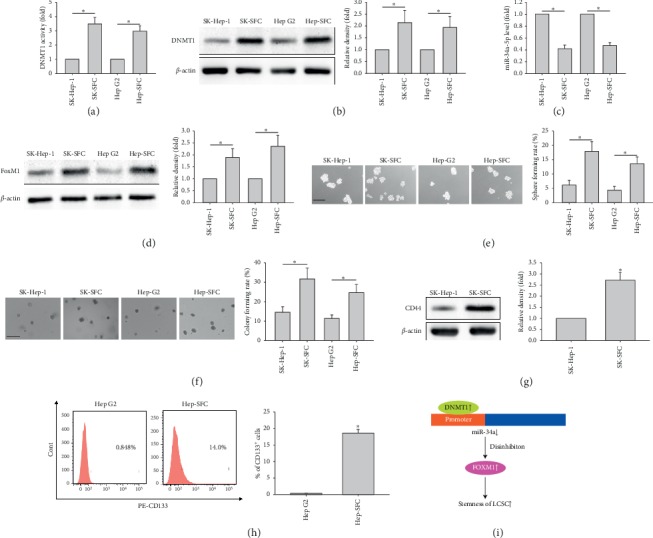
The DNMT1/miR-34a/FoxM1 axis is a novel target for stemness in LCSLCs from SK-Hep-1 and Hep G2 cells. (a, b) DNMT1 activities and protein amounts in SK-Hep-1 cells or Hep G2 cells and the corresponding spheres (served as LCSLCs). (c) miR-34a-5p levels. (d) FoxM1 expression levels in SK-Hep-1 and Hep G2 cells as well as respective LCSLCs, with *β*-actin as an internal control. ^*∗*^*P* < 0.05 vs. corresponding parent cells (*n* = 3). (e, f) Sphere and colony formation rates in SK-Hep-1 and Hep G2 cells as well as respective LCSLCs. (g) CD44 expression levels in SK-Hep-1 cells and LCSLCs. ^*∗*^*P* < 0.05 vs. SK-Hep-1 cells (*n* = 3). (h) CD133 expression levels in Hep G2 cells and LCSLCs. ^*∗*^*P* < 0.05 vs. Hep G2 cells (*n* = 3). (i) Schematic diagram of DNMT1/miR-34a/FOXM1 axis-mediated mechanism of acquisition and maintenance of stemness in liver cancer.

In order to assess the universality of the DNMT1/miR-34a/FoxM1 axis in LCSLCs, we selected two additional established liver cancer cell lines, including SK-Hep-1 and Hep G2 cells, to compare DNMT1 activities and protein amounts, miR-34a-5p levels, FoxM1 protein amounts, and stemness features between monolayer cells and the corresponding spheres, namely, LCSLCs. DNMT1 activity, DNMT1, and FoxM1 protein amounts, and sphere and colony formation capabilities in the corresponding LCSLCs were substantially increased, while miR-34a-5p levels were significantly decreased (Figures 8(a)–8(f)). We also assessed CD44 expression in SK-Hep-1 cells or CD133 expression in Hep G2 cells relative to the corresponding LCSLCs. The CSC marker (CD44 or CD133) was increased in the corresponding LCSLCs (Figures 8(g) and 8(h)). These results suggested that the DNMT1/miR-34a/FoxM1 axis promoted stemness in diverse LCSLCs. In addition, the DNMT1/miR-34a-5p/FoxM1 axis might be a novel target for stemness in LCSLCs.

## 4. Discussion

This study demonstrated that miR-34a promoter methylation by abnormal DNMT1 activation resulted in miR-34a downregulation in LCSCs compared with the corresponding liver cancer cells. Subsequently, upregulation of FOXM1 by disinhibition through miR-34a silencing promoted LCSC stemness and carcinogenicity in vitro and in vivo. These findings indicate that FOXM1 upregulation through the DNMT1/miR-34a signaling axis plays a key role in liver cancer progression, particularly in promoting the stemness of LCSCs.

Recently, silencing tumor repressors or suppressive microRNAs in precancerous cells by epigenetic regulation has attracted increasing attention [31]. DNMT1 is abnormally activated in tumor tissues and cancer stem cells and functions by catalyzing DNA methylation [32, 33]. In addition, DNMT1 is required for maintaining CSLC characteristics [33]. In breast cancer, epigenetic modifying agents, such as 5-azacytidine, could markedly reduce the CSC subpopulation [34]. Decitabine (5-aza-2′-deoxycytidine), which is more effective than 5-azacytidine, efficiently suppresses epithelial-mesenchymal transition (EMT) in NSCLC PC9 cells through an epigenetic-based therapeutic activity [35]. Our study provided evidence that DNMT1 was activated, and DNMT1 knockdown decreased stemness and carcinogenicity in LCSCs. These findings suggest that DNMT1 inhibition might represent a potential therapeutic strategy for human liver cancer by targeting LCSCs. Since DNMT1 is regulated by multiple factors (IL-6, STAT3, miR-148a, etc.), further research is required to understand why DNMT1 expression is elevated in LCSC [36–38].

Aberrant regulation of miRNAs is involved in controlling stemness in multiple cancers by modulating stemness-related genes [39–41]. MiR-34a is a tumor suppressor miRNA and is downregulated in many malignancies, for example, human liver cancer [10, 11]. Interestingly, miR-34a might be regulated by DNMT1 through promoter methylation in cancer cells or CSLCs [42]. We here showed that miR-34a were reduced in LCSCs compared with the corresponding liver cancer cells and decreased stemness features. In addition, miR-34a downregulation was involved in promoter methylation by activated DNMT1. Conversely, miR-34a did not alter DNMT1 activity and expression. This confirmed that epigenetic silencing of miR-34a promotes stemness features in LCSCs, highlighting the DNMT1/miR-34a axis might represent a promising approach in treating human liver cancer by targeting LCSCs.

Many studies have revealed that FOXM1, a carcinogenic transcriptional factor, has a critical function in the pathogenesis of various cancers, including liver cancer [43, 44]. However, the FOXM1-related upstream regulatory events in LCSCs are not fully understood. Our study clearly demonstrated that FOXM1 is a direct functional target of miR-34a in liver cancer cells. Knockdown and overexpression of FOXM1 could phenocopy and rescue the suppressive effects of miR-34a on stemness features in LCSCs, respectively, providing a rationale for stemness acquisition by miR-34a downregulation via promoter methylation by aberrant DNMT1 overexpression in LCSCs (Figure 8(i)).

## 5. Conclusion

In summary, given the critical role of stemness acquisition and maintenance in cancer pathogenesis and therapy, these findings have significant clinical implications. We demonstrated that DNMT1 overexpression promotes stemness via silencing of miR-34a (with the involvement of DNA methylation) and FOXM1 upregulation. This novel DNMT1/miR-34a/FOXM1 signaling-mediated mechanism of stemness acquisition and maintenance in liver cancer offers a new potential therapeutic strategy for the treatment of human liver cancer.

## Data Availability

The data sets generated and analyzed during the current study are available from the corresponding author on reasonable request.

## Abbreviations

CSC-CM: Cancer stem cell-conditioned culture medium
DNMT1: DNA methyltransferase 1
DAC: Decitabine
DMEM: Dulbecco's modified eagle's medium
FBS: Fetal bovine serum
LCSCs: Liver cancer stem cells
LCSLCs: Liver cancer stem-like cells
miR-34a: MicroRNA-34a
qRT-PCR: Quantitative reverse transcription polymerase chain reaction
NSCLC: Non-small-cell lung cancer.

## Supplementary Materials

Supplementary Materials Table S1: primer sequences for quantitative real-time RT-PCR. Table S2: primer sequences for methylation specific PCR. Table S3: comparison of tumor formation ability of HCSLC and parental MHCC97H cells in Balb/c-nu mice (*n* = 6). Supplementary Figure S1: comparing the volume of sphereiods between MHCC97H cells and LCSCs. Supplementary Figure S2: CD133+ cell percentages of LCSC during serial passage in sphere culture. ^*∗*^*P* < 0.05 vs. first generation of LCSC (*n* = 3). Supplementary Figure S3: effects of different DNMT1 shRNA on DNMT1 protein expression of MHCC97H derived LCSCs. ^*∗*^*P* < 0.05 vs. LCSC or sh NC (*n* = 3); ^#^*P* < 0.05 vs. shDNMT1 #1 (*n* = 3).
