# Supplementary material for: The DNMT1/miR-34a/FOXM1 Axis Contributes to Stemness of Liver Cancer Cells
Source: J Oncol. 2020 Apr 5;2020:8978930. doi: 10.1155/2020/8978930 (PMC7142390; doi:10.1155/2020/8978930)
Supplement: Supplementary Materials — Table S1: primer sequences for quantitative real-time RT-PCR. Table S2: primer sequences for methylation specific PCR. Table S3: comparison of tumor formation ability of HCSLC and parental MHCC97H cells in Balb/c-nu mice (n = 6). Supplementary Figure S1: comparing the volume of sphereiods between MHCC97H cells and LCSCs. Supplementary Figure S2: CD133+ cell percentages of LCSC during serial passage in sphere culture. ∗P < 0.05 vs. first generation of LCSC (n = 3). Supplementary Figure S3: effects of different DNMT1 shRNA on DNMT1 protein expression of MHCC97H derived LCSCs. ∗P < 0.05 vs. LCSC or sh NC (n = 3); #P < 0.05 vs. shDNMT1 #1 (n = 3). [file 8978930.f1.pdf]

Table S1. Primer sequences for quantitative real-time RT-PCR

Gene	Sequence(5'-3')
DNMT1	F: GGCTGAGATGAGGCAAAAAG<br>R: ACCAACTCGGTACAGGATGC
miR-34a-5p	F: TGGCAGTGTCTTAGCTGGTTGT<br>R: GCGAGCACAGAATTAATACGAC
CD133	F: ATGCTCTCAGCTCTCCCGC<br>R: TTCTGTCTGAGGCTGGCTTG
CD44	F: GCAAACACAACCTCTGGTCC<br>R: CCCACACCTTCTTCGACTGT
ALDH1	F: GCACGCCAGACTTACCTGTC<br>R: CCTCCTCAGTTGCAGGATTAAAG
Bmi1	F: CTGGTTGCCCATTGACAGCG<br>R: AAATCCCGGAAAGAGCAGCC
Sox2	F: GCCCTGCAGTACAACTCCAT<br>R: CTGATCATGTCCCGTAGGT
Oct4	F: GTGAGAGGCAACCTGGAGAG<br>R: GAATGGGACCGAGGAGTACA
FOXM1	F: ATACGTGGATTGAGGACCACT<br>R: TCCAATGTCAAGTAGCGGTTG
GAPDH	F: ACACTCACTCTTCTACCTTTG<br>R: CAAATTCATTGTCGTACCAG
U6	F: CTCGCTTCGGCAGCACA<br>R: AACGCTTCACGAATTTGCGT

Table S2. Primer sequences for methylation specific PCR

Gene	Sequence(5'-3')
miR-34a-5p-M	F: ATGAGGATTAGGATTTCGGAG<br>R: AACGCATAAAAACGACGACAA
miR-34a-5p-U	F: GGGGATGAGGATTAGGATTTT<br>R: ATAAAACTAAAACTCTACCTTCGCT

Table S3. Comparison of tumor formation ability of LCSCs and parental MHCC97H cells in Balb/c-nu mice (n = 6)

Cell Type	Cell Inoculated (n)	Tumor Incidence (n/4)	Latency (d)	Tumor volume (mm^3)	Tumor weight (g)
MHCC	10^5	6/6	23~48	659±76	0.54±0.15
HCSLC	10^5	6/6	6~12	1968±92*	1.75±0.25*

The control samples included the tumor volume and tumor weight of LCSCs xenografts. Comparisons were carried out for LCSCs derived from MHCC97H cells with control cells, $^*P< 0.05$.

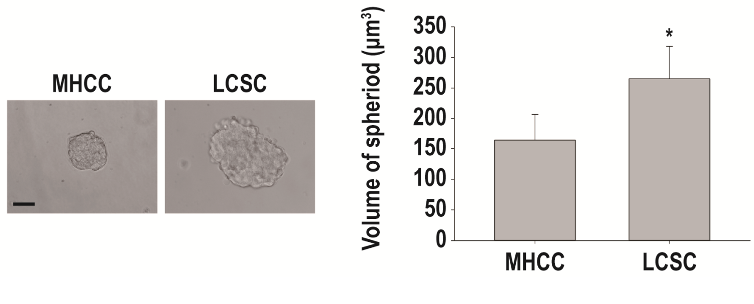

Supplementary Figure S1 Comparing the volume of sphereiods between MHCC97H cells and LCSCs.

$^*P<0.05$ vs MHCC97H cells (n=3).

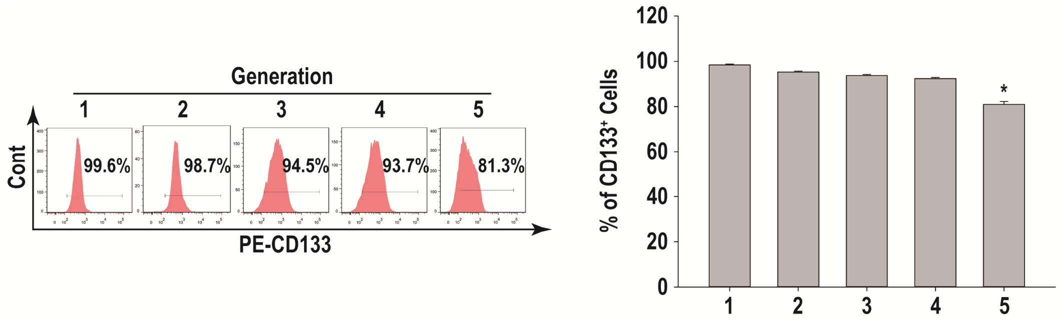

Supplementary Figure S2 CD133^{+} cell percentages of LCSC during serial passage in sphere culture.

**P*<0.05 vs First Generation of LCSC (n=3).

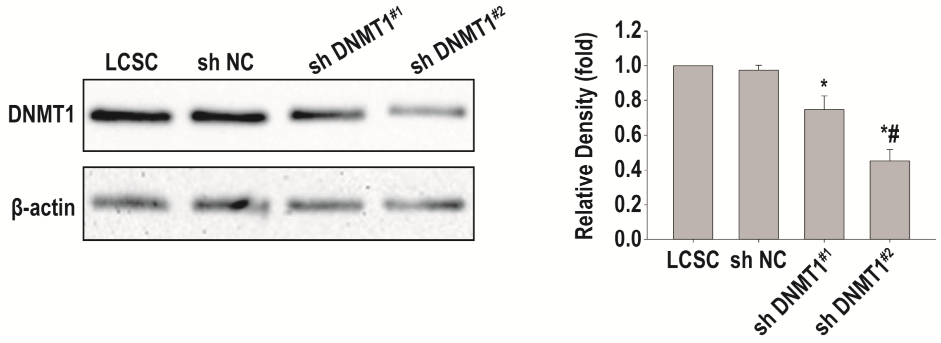

Supplementary Figure S3 Effects of different DNMT1 shRNA on DNMT1 protein expression of MHCC97H derived LCSCs

*P<0.05 vs LCSC or sh NC (n=3); $^{\#}$ P<0.05 vs sh DNMT1#1 (n=3).
